# The xylem as battleground for plant hosts and vascular wilt pathogens

**DOI:** 10.3389/fpls.2013.00097

**Published:** 2013-04-23

**Authors:** Koste A. Yadeta, Bart P. H. J. Thomma

**Affiliations:** ^1^Laboratory of Phytopathology, Wageningen UniversityWageningen, Netherlands; ^2^Centre for BioSystems GenomicsWageningen, Netherlands

**Keywords:** xylem, defense response, immunity, innate, pathogen, fungi, bacteria

## Abstract

Vascular wilts are among the most destructive plant diseases that occur in annual crops as well as in woody perennials. These diseases are generally caused by soil-borne bacteria, fungi, and oomycetes that infect through the roots and enter the water-conducting xylem vessels where they proliferate and obstruct the transportation of water and minerals. As a consequence, leaves wilt and die, which may lead to impairment of the whole plant and eventually to death of the plant. Cultural, chemical, and biological measures to control this group of plant pathogens are generally ineffective, and the most effective control strategy is the use of genetic resistance. Owing to the fact that vascular wilt pathogens live deep in the interior of their host plants, studies into the biology of vascular pathogens are complicated. However, to design novel strategies to combat vascular wilt diseases, understanding the (molecular) biology of vascular pathogens and the molecular mechanisms underlying plant defense against these pathogens is crucial. In this review, we discuss the current knowledge on interactions of vascular wilt pathogens with their host plants, with emphasis on host defense responses against this group of pathogens.

## INTRODUCTION

Plants are continuously confronted with potential pests and pathogens that include insects, nematodes, viruses, bacteria, fungi, and oomycetes. While many of these organisms evolved to infect aerial plant parts, such as leaves, stems, flowers, and fruits, others target below-ground organs, such as roots and tubers. Specific pathogens target the vascular system that is composed of xylem vessels, tracheary elements that transport water and minerals that are absorbed by the roots to the photosynthetic organs, and phloem elements, the living tissue that transports organic photosynthesis products. Paradoxically, although the phloem is rich in sugars, most vascular pathogens colonize the nutrient-poor xylem vessels. This may be explained by the accessibility of both types of vessel elements, as the phloem is characterized by living cells with a high osmotic pressure which makes penetration difficult, while the xylem is composed of dead tracheary elements with relatively low osmotic pressure. Consequently, phloem pathogens comprise rickettsias, spiroplasmas, and phytoplasmas that are introduced by vectors such as phloem feeding insects, or by cultural practices like grafting.

Xylem-invading pathogens comprise bacterial, fungal, and oomycete microorganisms that cause vascular wilt diseases. Vascular wilt pathogens are among the most destructive plant pathogens that can wipe out entire crops. Vascular wilt diseases occur worldwide and affect annual crops as well as woody perennials, thus not only impacting food and feed production, but also natural ecosystems. Most of the symptoms caused by vascular wilt pathogens develop in acropetal direction: from bottom to top. Epinasty is the primary disease symptom, followed by flaccidity, chlorosis, vascular browning, and necrostress tolerance when compared with non-stress tolerance when compared with non-sis of the terminal leaflets (Agrios, 2005). A large range of symptoms is caused by vascular wilt pathogens, and the same pathogen may cause different symptoms on different host plants. Depending on the pathogen species and the host, plants may become stunted, wilt partially or completely, and ultimately die. Plant death may occur within days to weeks or, in case of perennials, months to years (Purcell and Hopkins, 1996; Fradin and Thomma, 2006; Niño-Liu et al., 2006; Juzwik et al., 2008; Klosterman et al., 2009, 2011; Michielse and Rep, 2009; Genin, 2010; Janse and Obradovic, 2010; Harwood et al., 2011). Age, fitness, and the nutritional status of the host, environmental conditions, and virulence of the pathogen can all determine the speed and severity at which symptoms develop (Tjamos and Beckman, 1989; Hayward, 1991; Roncero et al., 2003; Niño-Liu et al., 2006; Chatterjee et al., 2008). In all cases where it is observed, wilting symptoms represent a transitory phase of the disease.

Vascular wilt pathogens generally overwinter in soil, plant debris, watercourses, or in insect vectors (Fradin and Thomma, 2006; Niño-Liu et al., 2006; Juzwik et al., 2008; Klosterman et al., 2009,^2011^; Michielse and Rep, 2009; Genin, 2010; Janse and Obradovic, 2010; Nadarasah and Stavrinides, 2011). While most vascular wilt pathogens are soil-borne and enter their hosts through the roots by penetration via wounds or cracks that appear at the sites of lateral root formation (Vicente et al., 2001; Di Pietro et al., 2003; Fradin and Thomma, 2006; Klosterman et al., 2009; Michielse and Rep, 2009; Genin, 2010), some enter leaves via natural openings such as stomata and hydathodes, such as the bacterial leaf blight pathogen of rice, *Xanthomonas oryzae* (Niño-Liu et al., 2006). Furthermore, some vascular wilt pathogens are delivered directly into the xylem by insect vectors that feed on xylem sap, such as *Xylella fastidiosa* bacteria that are transmitted by leafhoppers, or by chewing insects, such as *Ophiostoma* fungi that are transmitted by bark beetles (Purcell and Hopkins, 1996; Chatterjee et al., 2008; Moser et al., 2010; Nadarasah and Stavrinides, 2011). Regardless of the mechanism used by vascular wilt pathogens to enter their hosts, they subsequently colonize the xylem vessels where they proliferate (Tjamos and Beckman, 1989; Purcell and Hopkins, 1996; Agrios, 2005; Niño-Liu et al., 2006; Klosterman et al., 2009; Genin, 2010).

## CONTROL OF VASCULAR WILT DISEASES

Controlling vascular wilt pathogens is difficult for several reasons. First of all, no efficient treatments exist to cure infected plants, and growers generally have to remove them from their crops. Secondly, many vascular wilt pathogens are soil-borne and produce persistent resting structures that are able to survive for long periods of time in the absence of host plants. Thirdly, some of these pathogens can infect a broad range of host plants and as a consequence, cultural control measures such as crop rotation are generally not very effective. Resting structures are desirable targets for control by soil solarization and chemical fumigation. However, limitations in large-scale applicability and ban on chemical fumigants because of public health and environmental issues render these approaches unsuitable. Biological agents and organic soil amendments are used to control vascular wilt diseases (Tsuda et al., 2001; Spadaro and Gullino, 2005; Suárez-Estrella et al., 2007; Ji et al., 2008; Markakis et al., 2008). For instance, injection of the Dutch trig, a bio-control vaccine that contains conidia of a non-pathogenic strain of the vascular wilt fungus *Verticillium albo-atrum* isolate, into elm trees is used to induce the natural defense against Dutch elm disease caused by the fungi *O. ulmi* and *O. novo-ulmi* (Scheffer et al., 2008). However, since biological agents are often affected by biotic and abiotic factors, performance of bio-control microorganisms in the field is often inconsistent (Tsuda et al., 2001).

The most effective strategy to control vascular wilt diseases thus far is the use of genetic resistance in host plants. Due to the fact that vascular wilt pathogens live deep in the interior of their host plants, studies into the biology of vascular pathogens is complicated. However, their high economic impact, combined with the absence of curative treatments, justifies increased attention. The recent availability of a number of genome sequences of vascular pathogens has inspired novel research efforts to unravel the molecular basis of vascular wilt diseases (**Table 1**). To design novel strategies to combat vascular wilt diseases, understanding the (molecular) biology of vascular pathogens and the molecular mechanisms underlying plant defense against these pathogens is crucial.

**Table 1:** Publically available genome sequences of vascular wilt pathogens.

Organism	Species	Reference
Fungus	*Verticillium dahliae*	Klosterman et al. (2011)
	*Verticillium albo-atrum*	Klosterman et al. (2011)
	*Fusarium oxysporum* f. sp. *lycopersici*	Ma et al. (2010)
Bacterium	*Ralstonia solanacearum*	Salanoubat et al. (2002)
	*Xanthomonas oryzae *pv. *oryzae*	Lee et al. (2005)
	*Xanthomonas campestris *pv.\hb *campestris*	Qian et al. (2005)
	*Xylella fastidiosa*	Simpson et al. (2000)
	*Clavibacter michiganensis* ssp. *michiganensis*	Gartemann et al. (2008)
	*Erwinia amylovora*	Sebaihia et al. (2010), Smits et al. (2010), Powney et al. (2011)
Oomycete	*Pythium ultimum*	Lévesque et al. (2010)

## CAUSAL AGENTS OF VASCULAR WILT DISEASES

### FUNGAL VASCULAR WILT PATHOGENS

There are four fungal genera containing the major vascular wilt pathogens: *Ceratocystis* (vascular wilts of oak, cocoa, and eucalyptus), *Ophiostoma* (vascular wilts of elm trees), *Verticillium* (broad host range), and *Fusarium* (broad host range; Tjamos and Beckman, 1989; Agrios, 2005; Juzwik et al., 2008; Schumann and D’Arcy, 2010; Harwood et al., 2011; López-Escudero and Mercado-Blanco, 2011). In contrast to the other three genera, the vast majority of *Fusarium* vascular wilt pathogens all belong to a single species, *F. oxysporum*, which contains morphologically indistinguishable pathogenic and non-pathogenic strains (Lievens et al., 2008). The pathogenic strains cause vascular wilts or root rot in over 100 different host species (Di Pietro et al., 2003; Roncero et al., 2003; Michielse and Rep, 2009). Despite the broad host range of these species, individual strains typically infect only a single or a few hosts and are assigned to *formae speciales* (Michielse and Rep, 2009). Interestingly, it was experimentally demonstrated that the transfer of two lineage specific (LS) chromosomes from a tomato pathogenic *F. oxysporum* f. sp. *lycopersici* strain to a non-pathogenic strain converted the latter into a tomato pathogen, suggesting that host specificity within *F. oxysporum* may be determined by pathogenicity chromosomes (Ma et al., 2010). Such pathogenicity chromosomes have not been identified in vascular wilt pathogens of the *Verticillium* genus for which genome sequences have recently been determined as well (Klosterman et al., 2011).

Most fungal vascular wilt pathogens overwinter as resting structures in the soil or on dead host tissues. These include microsclerotia, chlamydospores, thick-walled mycelium, and spore-bearing coremia that all can survive for an extended period of time without losing viability. Compounds released from host plants, referred to as exudates, trigger germination of these resting structures. Except for *Ophiostoma* spp. and the oak wilt pathogen *Ceratocystis fagacearum* that are transmitted by beetles (Hayslett et al., 2008; Juzwik et al., 2008; Harwood et al., 2011), fungal vascular wilt pathogens enter their host plants through the roots. Following penetration, the fungi colonize the cortical cells from where hyphae migrate intercellularly toward the vascular parenchyma cells and invade the xylem vessels (Di Pietro et al., 2003; Klosterman et al., 2009; Schumann and D’Arcy, 2010; Nadarasah and Stavrinides, 2011). In the xylem, conidiospores are produced which are disseminated acropetally with xylem sap movement. Fungal vascular wilt pathogens are mostly restricted to xylem vessels, but once host tissues become necrotized also these are colonized and the fungus starts to produce resting structures which are released into the soil eventually (Di Pietro et al., 2003; Agrios, 2005; Fradin and Thomma, 2006).

### BACTERIAL VASCULAR WILT PATHOGENS

Seven bacterial genera contain vascular wilt pathogens: *Clavibacter* (causing ring rot of potato and bacterial canker and wilt of tomato), *Curtobacterium* (bacterial wilt of beans), *Erwinia* (bacterial wilt of cucurbits), *Pantoea* (stewart’s wilt of corn), *Ralstonia* (southern bacterial wilt of Solanaceous crops and Moko disease of banana), *Xanthomonas* (black rot of crucifers, bacterial blight of rice), and *Xylella* (Pierce’s disease of grape, citrus variegation chlorosis; Tjamos and Beckman, 1989; Agrios, 2005; Chatterjee et al., 2008; Schumann and D’Arcy, 2010; Nadarasah and Stavrinides, 2011; Roper, 2011). Bacterial vascular wilt pathogens overwinter in plant debris in soil, in seeds, in vegetative propagules, or in their insect vectors as dormant cells (Agrios, 2005). They enter host tissues only passively, via wounds, cracks, or natural openings such as stomata and hydathodes, while others are directly delivered into the xylem by insect vectors, such as *Xylella fastidiosa* by sharpshooter leafhoppers and spittlebugs, *Pantoea stewartii* by corn flea beetles and *E. tracheiphila* by cucumber beetles (Schumann and D’Arcy, 2010; Nadarasah and Stavrinides, 2011; Roper, 2011). After entrance, they rapidly multiply and invade the root cortex and vascular parenchyma intercellularly, from where they spread to the xylem vessels that are used as avenues for passive spread to aerial plant parts. During colonization, bacterial wilt pathogens degrade xylem cell wall components, parenchyma cells, and pit membranes, resulting in slimy masses of bacteria and cellular debris (Agrios, 2005; Schumann and D’Arcy, 2010).

### OOMYCETE VASCULAR WILT PATHOGENS

Only one oomycete genus, *Pythium*, contains vascular wilt pathogens. *Pythium* mainly infects seeds or seedlings in the soil, causing pre-emergence or post-emergence seedling damping-off disease, and young and juvenile plant tissues (Martin and Loper, 1999; Oliver et al., 2009). The genus *Pythium* comprises many complex species, most of which are plant pathogens, while others are saprophytes or animal parasites (Martin and Loper, 1999). *Pythium* species survive in soil or in organic substrates for long periods of time as dormant oospores; thick-walled sexual spores that can withstand harsh environmental conditions (Martin and Loper, 1999). Oospores germinate upon stimulation by exudates released from plants, and often produce a sporangium containing zoospores that are released and encysted after host contact. Alternatively, oospores produce germinating hyphae to penetrate the root epidermis, migrate through the cortex, endodermis, and parenchyma cells, and eventually invade the vascular stele causing typical damping-off symptoms (Rey et al., 1998).

## XYLEM STRUCTURE AND DEVELOPMENT

The xylem consists of distinct cells with special wall structures that allow efficient transport of water and solutes from the roots to upper plant parts. The xylem functions not only in long distance transport, but also provides physical strength to the plant. Xylem development occurs in two phases during which primary and secondary xylem is produced (Fukuda, 1997,^2004^; Ye, 2002; Zhang et al., 2011). Primary development involves the formation of primary xylem from procambium cells which are derived from the apical meristem. Procambium cells give rise to xylem precursor cells that eventually differentiate into treachery elements, xylem parenchyma cells or fiber cells; collectively called the xylem (Ye, 2002; Fukuda, 2004). Treachery elements, which consist of tracheid and vessel elements, are the main conductive tissues. While the xylem parenchyma cells are metabolically active and adapted for storage and transport, the xylem fiber cells together with treachery elements provide physical support (Nieminen et al., 2004). Following xylem differentiation, the treachery elements undergo cell elongation before the initiation of secondary xylem wall development (Ye, 2002; Fukuda, 2004; Nieminen et al., 2004; Zhang et al., 2011). The secondary xylem walls, which are derived from vascular cambium, are deposited onto the primary xylem walls (Fukuda, 1997; De Boer and Volkov, 2003). Secondary xylem is made of cellulose microfibrils, crystalline aggregates of linear polymers of D-glucopyranosyl residues linked in β-(1-4) conformation (Brett, 2000; Emons and Mulder, 2000). The secondary xylem walls are further impregnated with different polysaccharides, such as lignin, hemicellulose, pectin, and structural proteins that add strength and rigidity to the wall (Ye, 2002; Fukuda, 2004; Yokoyama and Nishitani, 2006). Subsequently, the secondary xylem walls are lignified, cross-linked, and eventually waterproofed by polymerization of the aromatic compound monolignol (Fukuda, 1996; De Boer and Volkov, 2003). The patterned secondary xylem walls provide physical strength to the treachery elements to withstand the negative pressure generated during transpiration and by the compressive pressure from surrounding cells (Ye, 2002; Nieminen et al., 2004; Choat and Pittermann, 2009; Zhang et al., 2011).

The final step of xylem development is the induction of programed cell death (PCD) that destroys the cellular contents of treachery elements, leaving behind hollow tube-like vessels through which water and nutrients flow (Fukuda, 1997; Zhang et al., 2011). The PCD is developmentally regulated and is strongly associated with secondary xylem wall formation (Fukuda, 2004). The vessel tubes are dedicated to the unrestricted water and solute movement throughout the plant and individual vessels are interconnected through small openings called pits (De Boer and Volkov, 2003; Choat and Pittermann, 2009). Pits between vessels typically have overarching secondary walls that form a bowl-shaped chamber, referred to as a border pit (De Boer and Volkov, 2003; Jansen et al., 2004). Border pit exists in pairs and contain a pit membrane at the center, which is formed from primary walls and the intervening middle lamella (De Boer and Volkov, 2003). The pit membrane is made of cellulose microfibrils embedded in polysaccharide matrix of hemicellulose and pectin (Tyree and Zimmermann, 2002; Pérez-Donoso et al., 2010). This fine mesh-like and tightly interlocked polysaccharide structure has minute openings through which water and solutes can move with a minimal resistance between vessels or to neighboring parenchyma cells (Choat and Pittermann, 2009). In angiosperm trees, the pit pore diameter varies between 5 and 20 nm (Choat et al., 2003,^2004^), thus acting as a safety mechanism to limit the spread of embolism within xylem vessels (Tyree and Zimmermann, 2002; De Boer and Volkov, 2003; Choat et al., 2008; Pérez-Donoso et al., 2010).

All vascular wilt pathogens have to breach the highly structured and rigid secondary xylem walls to enter the vessels. Also the pit membranes are a major barriers for vascular pathogens as vascular wilt pathogens are too large to pass pit membrane pores (Choat et al., 2003,^2004^). For example, the rod-shaped bacterium *Xylella fastidiosa* has a cell size of 0.25–0.5 μm in diameter (Mollenhauer and Hopkins, 1974), while the conidia of *Verticillium* species have a diameter of about 2.2 μm (Qin et al., 2008).

## THE XYLEM AS A NICHE FOR VASCULAR WILT PATHOGENS

The xylem is a nutritionally poor environment, which could be an important reason why only a limited number of plant pathogens are able to thrive in this environment. Possibly, vascular wilt pathogens exploit this niche to avoid competition with other microbes (McCully, 2001). However, as they reside in the xylem for the major part of their lifecycle, vascular pathogens need to be able to obtain all factors required for growth, reproduction, and survival.

### NUTRIENT COMPOSITION OF XYLEM SAP

Nitrate, sulfate, and phosphate are among the most abundant inorganic anions in the xylem sap, whereas calcium, potassium, magnesium, and manganese are the most predominate inorganic cations present in the xylem sap of oilseed rape (Nakamura et al., 2008). Although only in relatively low amounts, xylem sap also contains various carbohydrates, such as glucose, fructose, saccharose, maltose, raffinose, trehalose, and ribose (Alvarez et al., 2008; Nakamura et al., 2008; Fernandez-Garcia et al., 2011; Krishnan et al., 2011). Of these, glucose, fructose, and saccharose are predominant and are utilized as a carbon source for growth. Xylem sap furthermore contains various proteins, amino acids, and organic acids, which can also act as source of organic and inorganic nutrients (Alvarez et al., 2008; Nakamura et al., 2008; Fernandez-Garcia et al., 2011; Krishnan et al., 2011). For instance, the sulfur-containing amino acids methionine and cysteine can be used as a source of inorganic sulfur (Divon and Fluhr, 2007; Krishnan et al., 2011). Nevertheless, the quantities of the organic and inorganic compounds in the xylem sap are extremely low and fluctuate with day time, growth condition, and plant species (Siebrecht et al., 2003).

### NUTRIENT ACQUISITION BY VASCULAR WILT PATHOGENS

Vascular wilt pathogens satisfy their nutritional requirements by efficiently acquiring the scarce nutrients available in the xylem sap, by enzymatic digestion of host cell walls, by invading neighboring cells, or by inducing nutrient leakage from surrounding tissues (Divon et al., 2005; Möbius and Hertweck, 2009; Klosterman et al., 2011).

Nitrogen is one of the limiting nutrients in the xylem sap for vascular wilt pathogens (Divon et al., 2005). The preferred primary nitrogen sources for fungal pathogens, ammonia, glutamine, and glutamate (Marzluf, 1997; Divon et al., 2006), are scarce in xylem sap. In absence of primary nitrogen sources, fungi can utilize secondary nitrogen sources such as nitrate, nitrite, purines, amides, amino acids, and proteins (Marzluf, 1997; Divon et al., 2005,^2006^). GATA transcription factors, such as the *F. oxysporum* f. sp. *lycopersici* global nitrogen regulator (*FNR1*), are known to regulate utilization of secondary nitrogen sources (Marzluf, 1997; Divon and Fluhr, 2007; Bolton and Thomma, 2008; Donofrio et al., 2009). *FNR1* mutants grow normally on primary nitrogen sources, but fail to utilize secondary nitrogen sources such as amino acids, hypoxanthine, and uric acid (Divon et al., 2006). Disruption of *FNR1* not only affected virulence of *F. oxysporum* f. sp. *lycopersici* on tomato, but also regulation of three nitrogen acquisition genes, *Gap1*, *Mtd1*, and *Uricase* during growth *in planta*, suggesting that *FNR1* regulates the utilization of secondary nitrogen sources *in planta* (Divon et al., 2006).

Analysis of the whole genome sequences of *F. oxysporum* f. sp. *lycopersici*, *V. dahliae*, and *V. albo-atrum* showed that these genomes are enriched in genes that encode cell wall-degrading enzymes (CWDEs) that may be used for the enzymatic digestion of xylem walls and pit membranes (Ma et al., 2010; Klosterman et al., 2011). Also other vascular wilt pathogens are known as CWDE producers (Di Pietro et al., 2003; Jha et al., 2005; Sun et al., 2005; Fradin and Thomma, 2006; Michielse and Rep, 2009; Klosterman et al., 2011). While degrading cell wall components, these enzymes liberate sugars that may be used as nutrient sources.

Various vascular wilt pathogens produce high- and low-molecular weight phytotoxins during host colonization that have often been associated with wilt symptom development (Temple and Horgen, 2000; Wang et al., 2004; Palmer et al., 2005; Stipanovic et al., 2011; Zhou et al., 2012; Santhanam et al., 2013). As several phytotoxins disturb plant cell membrane integrity (Möbius and Hertweck, 2009), leakage of nutrients may occur from cells surrounding the xylem vessels that can be utilized by vascular wilt pathogens. For instance, two *Verticillium* necrosis- and ethylene inducing-like proteins, NLP1 and NLP2, were shown to display cytotoxic activity and differentially contribute to virulence on various host plant species, although the mechanism through which these NLPs contribute to virulence remains unclear (Zhou et al., 2012; Santhanam et al., 2013). Interestingly, compared with other ascomycete plant pathogens that typically contain up to three *NLP* genes, the *NLP* gene family is expanded in the *V. dahliae* genome (Klosterman et al., 2011; Santhanam et al., 2013). A similar expansion has been reported for the *F. oxysporum* genome, and it has been speculated that this expansion has contributed to their broad host range among dicotyledonous plant hosts (Ma et al., 2010; Klosterman et al., 2011). However, in addition to NLP1 and NLP2, none of the other *V. dahliae* NLPs were found to display cytotoxic activity, and their potential role in fungal virulence still remains enigmatic (Zhou et al., 2012; Santhanam et al., 2013).

Finally, although most vascular wilt pathogens are confined to xylem vessels, some of them degrade xylem vessel walls to colonize adjacent parenchyma cells (Agrios, 2005). These pathogens may obtain nutrition by parasitizing parenchyma cells.

## PLANT DEFENSE AGAINST VASCULAR WILT PATHOGENS

**FIGURE 1:**
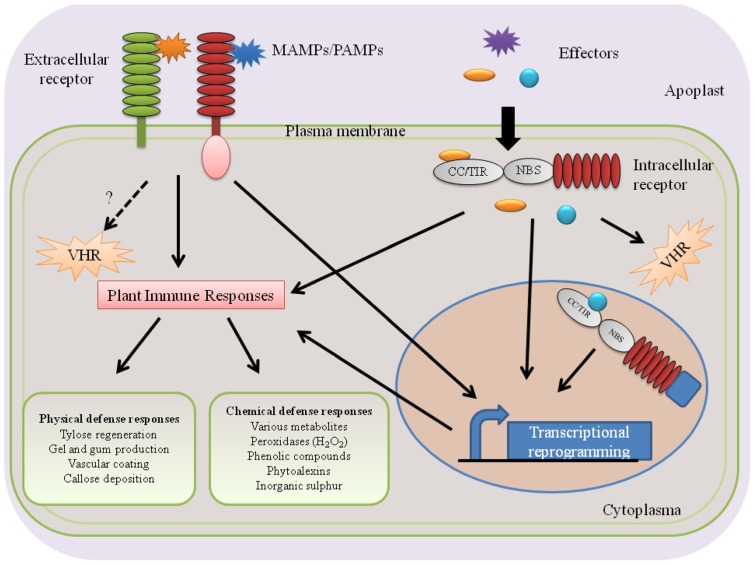
**Perception of vascular wilt pathogens and activation of subsequent plant immune responses**. Plants perceive PAMPs/MAMPs or effector proteins of vascular wilt pathogens using extracellular or intracellular receptors and activate immune responses in the xylem. The tomato receptor-like protein Ve1 and the rice receptor-like kinase Xa21 are examples of extracellular receptors that recognize *Verticillium* Ave1 and *Xanthomonas oryzae* pv. *oryzae* Xa21, respectively. Tomato I-2 and *Arabidopsis* RRS1-R are examples of intracellular NB–LRR-type receptors that perceive the *F. oxysporum* f. sp. *lycopersici* Avr2 effector and the R. solanacearum effector PopP2, respectively. Presumably, these processes take place in the parenchyma cells surrounding the xylem vessels.

Plants deploy two types of defenses against invading pathogens: pre-existing and inducible plant defense responses. The pre-existing defenses are constitutive and provide physical and chemical barriers against attempted host penetration. Once successful pathogens breach pre-existing defenses, they encounter a spectrum of inducible defense responses with microbe-associated molecular pattern (MAMP)-triggered immunity (MTI) and effector-triggered immunity (ETI) as two extreme ends (Jones and Dangl, 2006; Dodds and Rathjen, 2010). While MTI is activated upon recognition of conserved MAMPs, ETI is activated upon recognition of secreted effector proteins (**Figure 1**).

### PERCEPTION OF VASCULAR WILT PATHOGENS

**FIGURE 2:**
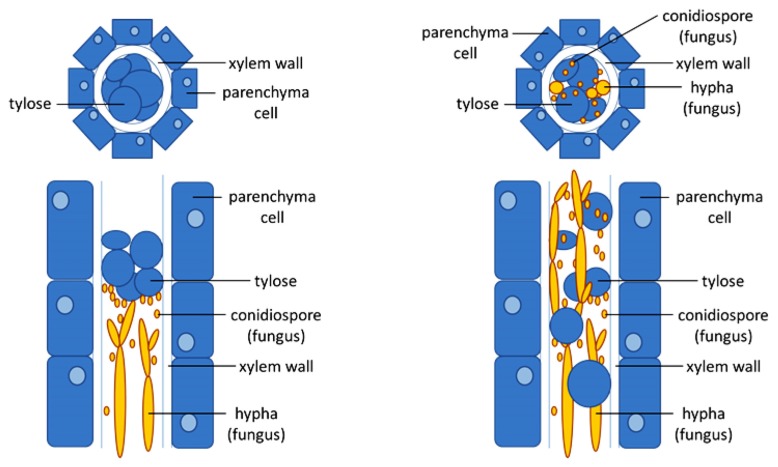
**Xylem occlusion limits pathogen growth in resistant plants.** Schematic drawing of cross-sections (top) or longitudinal sections (bottom) of a fungal-infected xylem vessel of a resistant (left) and a susceptible (right) plant. In the resistant plant, timely induction of the formation of tyloses, bubble-like outgrowth of the parenchyma contact cells surrounding the xylem vessels that protrude into the lumen of the vessel, are able to trap the fungus after which elimination can occur. As long as the number of vessels that is closed by the tyloses is limited, the host plant will not suffer from droughts stress. In the susceptible plant, tylose formation cannot trap the pathogen which is able to spread and further colonize the xylem.

In general, plants sense invading pathogens by using two types of receptors: extra- and intracellular receptors (**Figure 1**). While extracellular receptors recognize pathogen molecules on the cellular surface as well as damage-associated host molecules that are released as a consequence of pathogen activity, intracellular receptors recognize pathogen molecules that are delivered inside host cells (**Figure 1**). This extra- and intracellular receptor-mediated recognition of pathogen molecules [microbe-associated molecular patterns (MAMPs)/pathogen-associated molecular patterns (PAMPs), effectors] leads to the activation of plant innate immunity that wards off invading pathogens. Consequently, failure of a host plant to perceive invading pathogens leads to susceptibility and successful pathogen infections (**Figure 2**).

#### Extracellular plant receptors

Several MAMP receptors have been characterized, including *Arabidopsis* FLS2 (flagellin-sensitive 2), EFR (elongation factor Tu receptor), and CERK1 (chitin elicitor receptor kinase 1), and rice CEBiP (chitin elicitor binding protein). While FLS2 and EFR encode receptor-like kinases (RLKs) that recognize the bacterial MAMPs flg22 and EF-Tu, respectively (Gómez-Gómez et al., 2001; Kunze et al., 2004; Zipfel et al., 2006), CERK1 and CEBiP encode LysM domain-containing receptors that recognize chitin, the main constituent of fungal cell walls (Gómez-Gómez et al., 2001; Kunze et al., 2004; Zipfel et al., 2006). These MAMP receptors are considered to display a low degree of specificity and broadly act in pathogen defense.

Extracellular plant receptors that play a role in plant defense against specific vascular wilt pathogens have also been described. Rice Xa21 confers resistance against *Xanthomonas oryzae* pv. *oryzae* (*Xoo*; Song et al., 1995). Xa21 recognizes Ax21 (activator of Xa21), a type I-secreted sulfated protein (Song et al., 1995; Lee et al., 2009). Similar to *FLS2* and *EFR*, *Xa21* encodes a RLK (Song et al., 1995; Park et al., 2010b). Xa21 physically interacts with XB24, a protein with a C-terminal ATP synthase (ATPase) motif (Chen et al., 2010). XB24 promotes autophosphorylation of Ser/Thr residues on Xa21 through its ATPase activity, keeping Xa21 in an inactive state (Chen et al., 2010; Chen and Ronald, 2011). Upon Xa21-mediated Ax21 recognition, the Xa21 kinase becomes activated, triggering rice defense responses (Chen et al., 2010; Park et al., 2010b). XB25, plant-specific ankyrin-repeat family protein (Jiang et al., 2012), and BiP3, an endoplasmic reticulum chaperone protein (Park et al., 2010a), are also reported to be involved in Xa21-mediated rice immunity against *Xoo.*

Tomato Ve1 is another example of extracellular plant receptor that plays a role in xylem defense. Ve1 is an extracellular leucine-rich repeat (LRR) receptor-like protein (RLP; Kawchuk et al., 2001; Wang et al., 2008) that provides resistance against race 1 isolates of *V. dahliae* and *V. albo-atrum* (Fradin et al., 2009,^2011^). Interestingly, Fradin et al. (2011) have recently shown that interfamily transfer of *Ve1* gene to *Arabidopsis* confers resistance against race 1 isolates of *V. dahliae* and *V. albo-atrum*. Recently, the pathogen ligand that is perceived by Ve1 was identified as the Ave1 effector, a small (134 aa) effector protein with four cysteines that is required for full virulence on tomato plants lacking Ve1 (De Jonge et al., 2012). Ave1 is homologous to a widespread family of plant natriuretic peptides, mobile signaling molecules that play a role in the regulation of water and ion homeostasis, and it was suggested that *Ave1* was acquired by *Verticillium* through horizontal gene transfer from plants (De Jonge et al., 2012). Ave1 homologs are found in a few other plant pathogens, including the vascular wilt fungus *F. oxysporum* f. sp. *lycopersici*, but a role in virulence has not yet been demonstrated for these homologs (De Jonge et al., 2012). Intriguingly, the Ave1 homolog from *F. oxysporum* f. sp. *lycopersici* is recognized by Ve1 upon transient co-expression in tobacco, and Ve1 was found to mediate resistance toward *F. oxysporum* f. sp. *lycopersici* in tomato (De Jonge et al., 2012).

Recently, Zhang et al. (2013) have reported the presence of a functional *Ve1* ortholog in the tobacco species *Nicotiana glutinosa*, as *Ave1* expression in *N. glutinosa* causes an hypersensitive response (HR), rapid and localized cell death of plant tissue surrounding the site where recognition of pathogen effectors by host immune receptors occurs. Furthermore, *N. glutinosa* shows resistance against race 1 *V. dahliae* that is compromised upon inoculation with an *Ave1* deletion mutant of a race 1 *V. dahliae* isolate (Zhang et al., 2013).

#### Intracellular plant receptors

Also intracellular plant receptors that mediate plant defense against xylem-invading pathogens have been characterized. The tomato *I-2* gene is an intracellular receptor that contributes to resistance against race 2 isolates of *F. oxysporum* f. sp. *lycopersici* (Huang and Lindhout, 1997; Takken and Rep, 2010). It encodes a cytoplasmic CC (coiled-coil)–NBS (nucleotide-binding site)–LRR receptor protein that recognizes the effector protein Avr2, which was initially identified from the xylem sap of tomato infected by *F. oxysporum* f. sp. *lycopersici* and is taken up by tomato cells (Houterman et al., 2007,^2009^; Takken and Rep, 2010).

The *Arabidopsis* RRS1-R resistance protein is an intracellular plant receptor that confers resistance against *R. solanacearum*. RRS1-R encodes a TIR (Toll/interleukin1 receptor)–NBS–LRR R-protein and contains a C-terminal nuclear localization signal (NLS) and a WRKY domain (Deslandes et al., 2002). It recognizes the *R. solanacearum* type III-secreted effector protein PopP2 (Deslandes et al., 2003). RRS1-R physically interacts with the effector PopP2 (Deslandes et al., 2003). RRS1-R requires RD19, a cysteine protease that also binds to PopP2 (Deslandes et al., 2003; Bernoux et al., 2008). RD19 is localized in the vacuole in absence of PopP2 and re-localizes to the nucleus in the presence of PopP2 (Deslandes et al., 2003; Bernoux et al., 2008). However, no direct interaction between RRS1-R and RD19 has been reported so far. Thus, the current notion is that RRS1-R potentially recognizes the RD19/PopP2 complex in the nucleus and activates the *Arabidopsis* ETI against *R. solanacearum*.

### PLANT DEFENSE RESPONSES IN THE XYLEM

Comparison of the transcriptional changes in tomato induced by *Cladosporium fulvum*, a fungal foliar pathogen that causes leaf mold of tomato, with those induced by the vascular wilt pathogen *V. dahliae* revealed that the *Cladosporium fulvum*-induced transcriptional changes showed little overlap with those induced by *V. dahliae* (van Esse et al., 2009). Moreover, within the subset of genes that are regulated by both pathogens, many genes showed inverse regulation (van Esse et al., 2009).

Recognition of vascular wilt pathogens mediated by either extracellular or intracellular receptors leads to the activation of defense responses in the xylem vessels. These comprise physical defense responses which halt or contain the pathogen from further spread in the xylem vessels, and chemical defense responses that kill the pathogen or inhibit its growth (**Figure 2**).

A common defense mechanism in xylem vessels against vascular wilt pathogens is the formation of tyloses (Beckman, 1964; Talboys, 1972; Rahman et al., 1999; Fradin and Thomma, 2006). Tyloses are outgrowths of vessel-associated parenchyma cells which protrude into the xylem vessel through pits and block the spread of pathogens (Beckman, 1964; Talboys, 1972; Grimault et al., 1994; Agrios, 2005). They are formed during both compatible and incompatible interactions between the host and vascular wilt pathogens, although the time and extent of tylose formation significantly differs (**Figure 2**). Tyloses form much faster and more extensively in resistant plants when compared to susceptible plants (Grimault et al., 1994; Fradin and Thomma, 2006).

Often, the generation of tyloses is associated with the production of gels and gums around the differentiated tylose (Clérivet et al., 2000). Using immuno-gold labeling, strong accumulation of pectin-rich materials around the parenchyma cells, pit membrane, and the newly emerging tylose was observed in the xylem vessels of *Platanus acerifolia* cultivar infected by *Ceratocystis fimbriata* f. sp. *platani* (Clérivet et al., 2000). Plants potentially accumulate these pectin-rich gels and gums around tyloses to completely seal off a xylem vessel to prevent the vascular wilt pathogen from spread to adjacent xylem vessels (Rahman et al., 1999). However, complete sealing of xylem vessels can be disadvantageous for the plant as well. If tylose formation affects too many vessels and no new vessels are formed, tylose formation can result in drought stress (Fradin and Thomma, 2006).

Another typical symptom of *Verticillium* infection is vein clearing. Based on infections of *V. longisporum* on *Arabidopsis* and *Brassica napus*, it was recently reported that this vein clearing is caused by *Verticillium*-induced transdifferentiation of chloroplast-containing bundle sheath cells to functional xylem elements (Reusche et al., 2012). In addition, it was shown that infected *Arabidopsis* wild-type plants display enhanced drought stress tolerance compared with non-infected plants, suggesting that *Verticillium* infection activates a tissue-specific developmental program that compensates for compromised water transport (Reusche et al., 2012).

Another physical defense response observed during xylem colonization is vascular coating. A quick vascular wall coating around the initially infected and the adjacent xylem vessels, infusing the pit membrane and primary walls was observed in resistant chili pepper inoculated with *R. solanacearum*, whereas the xylem wall coating was not observed in susceptible chili pepper (Rahman et al., 1999). Similar coating of xylem parenchyma cells induced by *V. albo-atrum* was reported in tomato (Street et al., 1986) and alfalfa (Newcombe and Robb, 1988), indicating that infusion of pit membranes, primary walls and parenchyma cells with coating materials could prevent lateral and vertical spreading of vascular wilt pathogens in the xylem vessels. Furthermore, callose deposition and swelling of the primary walls of the xylem vessels was reported during the interaction of *R. solanacearum* with chili pepper (Rahman et al., 1999). Previously, a similar deposition of callose in resistant and susceptible tomato infected with *F. oxysporum* f. sp. *lycopersici* was reported (Beckman et al., 1982). However, the resistant cultivar maintains a stronger level of callose deposition during the course of the infection than the susceptible cultivar (Beckman et al., 1982). This high level deposition of callose in the resistant cultivar around the initially infected cells could inhibit pathogens from further spread.

Xylem colonization by *Xanthomonas campestris* pv. *campestris* has been reported to activate vascular immunity that triggers an HR, referred to as vascular HR (Xu et al., 2008). Vascular immunity was proposed based on the fact that AvrAC_Xcc8004_ (also referred to as XopAC), a type III effector protein of *Xanthomonas campestris* pv. *campestris* that confers avirulence in *Arabidopsis* ecotype Col-0, provides resistance when exclusively targeted to the vascular system (Xu et al., 2008). Infiltration of AvrAC_Xcc8004_ into leaf mesophyll tissue of Col-0 did not trigger resistance against *Xanthomonas*, implying that *AvrAC*_Xcc8004_-mediated activation of defense (vascular immunity) occurs in the xylem. Castañeda et al. (2005) previously reported that the *Xanthomonas campestris* pv. *campestris* effector protein AvrXccFM elicits vascular HR on Florida mustard seedlings. It is, however, important to note that unlike the HR occurring in leaf mesophyll cells, vascular HR is difficult to score (Castañeda et al., 2005; Xu et al., 2008).

*WAT1* (*Walls Are Thin1*), which is involved in secondary cell wall deposition, is also implicated in vascular immunity (Denancé et al., 2012). *WAT1* mutant *Arabidopsis* plants are resistant to bacterial and fungal vascular wilt pathogens but not to foliar pathogens (Denancé et al., 2012). In leaf inoculation assays, *wat1* provides resistance to *R. solanacearum *and *Xanthomonas campestris* pv. *campestris* only when directly injected into the vascular system, but not when injected into mesophyll tissues (Denancé et al., 2012), demonstrating the tissue-specific immune response. Likely, *wat1* resistance involves root-localized metabolic channeling away from indole metabolites to salicylic acid (Denancé et al., 2012).

*Arabidopsis* AHL19, an AT-hook DNA binding protein, provides enhanced resistance to three plant pathogenic *Verticillium* spp. but not to the foliar pathogens *Botrytis cinerea*, *Plectosphaerella cucumerina*, and *Alternaria brassicicola* and enhanced susceptibility to *Pseudomonas syringae* pv. *tomato*, suggesting a role as positive regulator of xylem-specific plant immunity (Yadeta et al., 2011).

Xylem infection causes drastic metabolic changes in xylem parenchyma cells, which are located adjacent to the infected vessels. These metabolic changes lead to the accumulation of different proteins and secondary metabolites in the xylem sap. Some of the proteins and secondary metabolites that accumulate in the xylem sap during xylem colonization include PR-1, PR-2, PR-3, PR-4, PR-5, peroxidases, proteases, xyloglucan-endotransglycosylase (XET), and xyloglucan-specific endoglucanase inhibitor protein (XEGIP), phenols, phytoalexins, and lignin-like compounds (Cooper et al., 1996; Hilaire et al., 2001; Rep et al., 2002,^2003^; Williams et al., 2002; Houterman et al., 2007; Basha et al., 2010; Gayoso et al., 2010). These compounds are known to contribute directly or indirectly to plant defense. The PR-1, PR-2, PR-3, and PR-5 were also among the proteins abundantly accumulated in xylem sap during compatible interaction between *Fusarium* and tomato (Rep et al., 2002; Houterman et al., 2007). For instance, PR-2 (β-1, 3-glucanase) and PR-3 (chitinase) hydrolyze the fungal cell wall component β-1,3-glucan and chitin, respectively (Leubner-Metzger and Meins, 1999; van Loon et al., 2006). In addition, antimicrobial activity of PR-5 proteins has also been demonstrated toward multiple pathogens (van Loon et al., 2006), implying that the presence of these proteins in xylem sap could inhibit or slow down the growth of the fungal vascular wilt pathogens in the xylem vessels.

Peroxidases are among the abundantly accumulated enzymes in xylem sap during host colonization of vascular wilt pathogens. The cationic peroxidase, PO-C1, accumulates in the cytoplasm, the primary and secondary walls of the xylem parenchyma, and lumen cells during incompatible interactions between *Xoo* and rice (Hilaire et al., 2001). Peroxidases are heme-containing enzymes that catalyze the oxidation of different substrates using hydrogen peroxides as an electron acceptor (Gayoso et al., 2010). Peroxidases are known to be involved in the production of reactive oxygen species through their enzymatic activity and reactive oxygen species are toxic compounds that can eliminate vascular wilt pathogens. Furthermore, peroxidases are implicated in the polymerization of cell wall compounds, lignin and suberin biosynthesis, and regulation of hydrogen peroxide levels, which all can contribute to defense (Hilaire et al., 2001; Passardi et al., 2005).

Plants accumulate different phenolic compounds in the xylem in response to infection. Olive trees accumulate phenols such as rutin, oleuropein, luteolin-7-glucoside, and tyrosol at the site of *V. dahliae* infection that were shown to have a toxic effect on *V. dahliae* (Báidez et al., 2007). Interestingly, exogenous treatment of Dutch elm trees with phenolic compounds induces accumulation of suberin-like compounds in the xylem tissue and thereby increases resistance to *O. novo-ulmi* (Martïn et al., 2008). This indicates that, in addition to direct toxicity, phenolic compounds could also activate other defense responses against vascular wilt pathogens.

Plants employ not only complex organic phytoalexins as defense mechanism against vascular wilt pathogens, but also employ inorganic compounds such as elemental sulfur and sulfur-containing inorganic compounds (Williams et al., 2002; Cooper and Williams, 2004). During an incompatible interaction between *V. dahliae* and tomato elemental sulfur mainly accumulates in xylem parenchyma cells, xylem vessel walls, and around the vascular occluding gels (Williams et al., 2002). Similar accumulation of elemental sulfur has been observed in an incompatible interaction between *V. dahliae* and cacao (*Theobroma cacao*) or cotton (Cooper et al., 1996; Cooper and Williams, 2004). The accumulation of inorganic sulfur specifically in xylem vessel walls and around the vascular occluding gels might suggest its role in eliminating vascular wilt pathogens that are arrested by physical defense responses.

Overall, chemical defense responses play major roles in xylem defense. Some chemical compounds accumulated in xylem sap after infection modulate the morphology of xylem tissue and by doing so inhibit vertical and lateral colonization of the pathogens, whereas other compounds accumulate during xylem infection have antimicrobial activity and can eliminate vascular wilt pathogens contained by the physical defense responses.

## CONCLUSION

Vascular wilt pathogens have adapted to thrive in the xylem, which is known as a nutrient-poor niche, causing vascular wilt diseases on hundreds of plant species. Recognition of vascular wilt pathogens by both extra- and intracellular plant receptors triggers plant innate immune responses that comprise physical and chemical defenses. Both types of defense responses occur in the xylem vessels in a coordinated manner, where physical defense responses mainly prevent the pathogens from spreading in the xylem vessels and chemical defense responses kill the pathogen or inhibit its growth.

Currently, little is known about the interaction between vascular wilt pathogens and their hosts. As this interaction takes place in xylem vessels which are located deep in the plant interior, the molecular basis underlying the interaction between vascular wilt pathogens and their hosts remains largely obscure. Genetic resistance is the best strategy for controlling vascular wilt pathogens. To develop genetic resistance, however, a deeper understanding of the host defense mechanisms as well as the biology, evolution and pathogenicity of vascular wilt pathogens is required.

### VASCULAR WILT PATHOGENS INDUCE DROUGHT STRESS

Wilting of plant parts as a consequence of xylem dysfunction is the most conspicuous symptom of vascular wilt disease. Daugherty et al. (2010) have nicely demonstrated using stable carbon isotope labeling that *Xylella fastidiosa* induces drought stress in alfalfa. Many factors can contribute to xylem occlusion, such as high- and low-molecular weight polysaccharides secreted by vascular wilt pathogens during xylem colonization and the presence of pathogen biomass (bacterial cells and fungal and oomycete mycelium and spores) in the xylem vessels. However, also plant defense responses can contribute to xylem occlusion, such as tyloses that are formed by the parenchyma cells and gum and gels that are secreted (Fradin and Thomma, 2006; Klosterman et al., 2009; Beattie, 2011). Embolism (the formation of air bubbles) in xylem vessels is another factor that can reduce the hydraulic conductivity of the xylem. Pérez-Donoso et al. (2007) have demonstrated using magnetic resonance imaging that *Xylella fastidiosa*-infected grape displayed early occurrence of embolism, which correlated with decreased xylem conductivity and drought stress. Although several research reports identify a correlation between xylem infection and drought stress, a recent report revealed enhanced drought tolerance in *Arabidopsis* upon *Verticillium* infection (Reusche et al., 2012). *Arabidopsis* plants infected with *V. longisporum* exhibited increased de novo xylem formation with newly transdifferentiated xylem vessels that were able to compensate for the occluded ones. Consequently, the plants showed higher drought stress tolerance when compared with non-infected plants.

## Conflict of Interest Statement

The authors declare that the research was conducted in the absence of any commercial or financial relationships that could be construed as a potential conflict of interest.

## References

[B1] Agrios G. N. (2005). *Plant Pathology*. Burlington, MA: Elsevier Academic Press

[B2] Alvarez S. Marsh E. L. Schroeder S. G. Schachtman D. P. (2008). Metabolomic and proteomic changes in the xylem sap of maize under drought. *Plant Cell Environ.* 31 325–340 18088330 10.1111/j.1365-3040.2007.01770.x

[B3] Báidez A. G. Gómez P. Del Rïo J. A Ortuño A. (2007). Dysfunctionality of the xylem in *Olea europaea* L. plants associated with the infection process by *Verticillium dahliae* Kleb. role of phenolic compounds in plant defense mechanism.* J. Agric. Food Chem.* 55 3373–3377 10.1021/jf063166d 17394331

[B4] Basha S. Mazhar H. Vasanthaiah H. (2010). Proteomics approach to identify unique xylem sap proteins in Pierce’s disease-tolerant *Vitis* species. *Appl. Biochem. Biotechnol.* 160 932–944 19412582 10.1007/s12010-009-8620-1

[B5] Beattie G. A. (2011). Water relations in the interaction of foliar bacterial pathogens with plants. *Annu. Rev. Phytopathol.* 49 533–555 21438680 10.1146/annurev-phyto-073009-114436

[B6] Beckman C. H. (1964). Host responses to vascular infection. *Annu. Rev. Phytopathol.* 2 231–252

[B7] Beckman C. H. Mueller W. C. Tessier B. J. Harrison N. A. (1982). Recognition and callose deposition in response to vascular infection in *Fusarium* wilt-resistant or susceptible tomato plants. *Physiol. Plant Pathol.* 20 1–10

[B8] Bernoux M. Timmers T. Jauneau A. Brière C. de Wit P. J. G. M. Marco Y. (2008). RD19, an *Arabidopsis* cysteine protease required for RRS1-R-mediated resistance, is relocalized to the nucleus by the *Ralstonia solanacearum* PopP2 effector. *Plant Cell* 20 2252–2264 18708476 10.1105/tpc.108.058685 PMC2553607

[B9] Bolton M. D Thomma B. P. H. J. (2008). The complexity of nitrogen metabolism and nitrogen-regulated gene expression in plant pathogenic fungi. *Physiol. Mol. Plant Pathol.* 72 104–110

[B10] Brett C. T. (2000). Cellulose microfibrils in plants: biosynthesis, deposition, and integration into the cell wall. *Int. Rev. Cytol.* 199 161–199 10874579 10.1016/s0074-7696(00)99004-1

[B11] Castañeda A. Reddy J. D. El-Yacoubi B. Gabriel D. W. (2005). Mutagenesis of all eight avr genes in *Xanthomonas campestris* pv. *campestris* had no detected effect on pathogenicity, but one avr gene affected race specificity. *Mol. Plant Microbe Interact.* 18 1306–1317 10.1094/MPMI-18-1306 16478050

[B12] Chatterjee S. Almeida R. P. P. Lindow S. (2008). Living in two worlds: the plant and insect lifestyles of *Xylella fastidiosa*. *Annu. Rev. Phytopathol.* 46 243–271 18422428 10.1146/annurev.phyto.45.062806.094342

[B13] Chen X. Chern M. Canlas P. E. Ruan D. Jiang C. Ronald P. C. (2010). An ATPase promotes autophosphorylation of the pattern recognition receptor XA21 and inhibits XA21-mediated immunity. *Proc. Natl. Acad. Sci. U.S.A.* 107 8029–8034 20385831 10.1073/pnas.0912311107 PMC2867851

[B14] Chen X. Ronald P. C. (2011). Innate immunity in rice. *Trends Plant Sci.* 16 451–459 21602092 10.1016/j.tplants.2011.04.003 PMC3152591

[B15] Choat B. Ball M. Luly J. Holtum J. (2003). Pit membrane porosity and water stress-induced cavitation in four co-existing dry rainforest tree species. *Plant Physiol.* 131 41–48 12529513 10.1104/pp.014100 PMC166785

[B16] Choat B. Cobb A. R. Jansen S. (2008). Structure and function of bordered pits: new discoveries and impacts on whole-plant hydraulic function. *New Phytol.* 177 608–626 18086228 10.1111/j.1469-8137.2007.02317.x

[B17] Choat B. Jansen S. Zwieniecki M. A. Smets E. Holbrook N. M. (2004). Changes in pit membrane porosity due to deflection and stretching: the role of vestured pits. *J. Exp. Bot.* 55 1569–1575 15181107 10.1093/jxb/erh173

[B18] Choat B. Pittermann J. (2009). New insights into bordered pit structure and cavitation resistance in angiosperms and conifers. *New Phytol.* 182 557–560 19422544 10.1111/j.1469-8137.2009.02847.x

[B19] Clérivet A. Déon V. Alami I. Lopez F. Geiger J. P. Nicole M. (2000). Tyloses and gels associated with cellulose accumulation in vessels are responses of plane tree seedlings (*Platanus* × *acerifolia*) to the vascular fungus *Ceratocystis fimbriata* f. sp *platani*. *Trees Struct. Funct.* 15 25–31

[B20] Cooper R. M. Resende M. L. V. Flood J. Rowan M. G. Beale M. H. Potter U. (1996). Detection and cellular localization of elemental sulphur in disease-resistant genotypes of *Theobroma cacao*. *Nature* 379 159–162

[B21] Cooper R. M. Williams J. S. (2004). Elemental sulphur as an induced antifungal substance in plant defence. *J. Exp. Bot.* 55 1947–1953 15181110 10.1093/jxb/erh179

[B22] Daugherty M. Lopes J. S. Almeida R. P. (2010). Strain-specific alfalfa water stress induced by *Xylella fastidiosa*. *Eur. J. Plant Pathol.* 127 333–340

[B23] De Boer A. H. Volkov V. (2003). Logistics of water and salt transport through the plant: structure and functioning of the xylem. *Plant Cell Environ.* 26 87–101

[B24] De Jonge R. Van Esse H. P. Maruthachalam K. Bolton M. D. Santhanam P. Saber M. K. (2012). Tomato immune receptor Ve1 recognizes effector of multiple fungal pathogens uncovered by genome and RNA sequencing. *Proc. Natl. Acad. Sci. U.S.A.* 109 5110–5115 22416119 10.1073/pnas.1119623109 PMC3323992

[B25] Denancé N. Ranocha P. Oria N. Barlet X. Rivière M.-P. Yadeta K. A. (2012). *Arabidopsis* wat1 (walls are thin1)-mediated resistance to the bacterial vascular pathogen, *Ralstonia solanacearum*, is accompanied by cross-regulation of salicylic acid and tryptophan metabolism. *Plant J.* 10.1111/tpj.12027 [Epub ahead of print]. 22978675

[B26] Deslandes L. Olivier J. Peeters N. Feng D. X. Khounlotham M. Boucher C. (2003). Physical interaction between RRS1-R, a protein conferring resistance to bacterial wilt, and PopP2, a type III effector targeted to the plant nucleus. *Proc. Natl. Acad. Sci. U.S.A.* 100 8024–8029 12788974 10.1073/pnas.1230660100 PMC164706

[B27] Deslandes L. Olivier J. Theulières F. Hirsch J. Feng D. X. Bittner-Eddy P. (2002). Resistance to *Ralstonia solanacearum* in *Arabidopsis thaliana* is conferred by the recessive RRS1-R gene, a member of a novel family of resistance genes. *Proc. Natl. Acad. Sci. U.S.A.* 99 2404–2409 11842188 10.1073/pnas.032485099 PMC122377

[B28] Di Pietro A. Madrid M. P. Caracuel Z. Delgado-Jarana J Roncero M. I. G. (2003). *Fusarium oxysporum*: exploring the molecular arsenal of a vascular wilt fungus. *Mol. Plant Pathol.* 4 315–325 20569392 10.1046/j.1364-3703.2003.00180.x

[B29] Divon H. H. Fluhr R. (2007). Nutrition acquisition strategies during fungal infection of plants. *FEMS Microbiol. Lett.* 266 65–74 17083369 10.1111/j.1574-6968.2006.00504.x

[B30] Divon H. H. Rothan-Denoyes B. Davydov O. Di Pietro A. Fluhr R. (2005). Nitrogen-responsive genes are differentially regulated in planta during *Fusarium oxysporum* f. sp. *lycopersici* infection. *Mol. Plant Pathol.* 6 459–470 10.1111/j.1364-3703.2005.00297.x 20565671

[B31] Divon H. H. Ziv C. Davydov O. Yarden O. Fluhr R. (2006). The global nitrogen regulator, FNR1, regulates fungal nutrition-genes and fitness during *Fusarium oxysporum* pathogenesis. *Mol. Plant Pathol.* 7 485–497 20507463 10.1111/j.1364-3703.2006.00354.x

[B32] Dodds P. N. Rathjen J. P. (2010). Plant immunity: towards an integrated view of plant–pathogen interactions. *Nat. Rev. Genet.* 11 539–548 20585331 10.1038/nrg2812

[B33] Donofrio N. M. Mitchell T. K. Dean R. A. Wang G.-L. Valent B. (2009). “The significance of nitrogen regulation, source and availability on the interaction between rice and rice blast,” in *Advances in Genetics, Genomics and Control of Rice Blast Disease*, edsWang G.-L. Valent B. (Dordrecht: Springer) 59–72

[B34] Emons A. M. C. Mulder B. M. (2000). How the deposition of cellulose microfibrils builds cell wall architecture. *Trends Plant Sci.* 5 35–40 10637660 10.1016/s1360-1385(99)01507-1

[B35] Fernandez-Garcia N. Hernandez M. Casado-Vela J. Bru R. Elortza F. Hedden P. (2011). Changes to the proteome and targeted metabolites of xylem sap in*Brassica oleracea* in response to salt stress. *Plant Cell Environ.* 34 821–836 21276013 10.1111/j.1365-3040.2011.02285.x

[B36] Fradin E. F. Abd-El-Haliem A. Masini L. van den Berg G. C. M. Joosten M. H. A. J. Thomma B. P. H. J. (2011). Interfamily transfer of tomato ve1 mediates *Verticillium* resistance in *Arabidopsis*. *Plant Physiol.* 156 2255–2265 21617027 10.1104/pp.111.180067 PMC3149960

[B37] Fradin E. F Thomma B. P. H. J. (2006). Physiology and molecular aspects of *Verticillium* wilt diseases caused by *V. dahliae* and *V. albo-atrum*. *Mol. Plant Pathol.* 7 71–86 20507429 10.1111/j.1364-3703.2006.00323.x

[B38] Fradin E. F. Zhang Z. Ayala J. C. J. Castroverde C. D. M. Nazar R. N. Robb J. (2009). Genetic dissection of *Verticillium* wilt resistance mediated by tomato ve1. *Plant Physiol.* 150 320–332 19321708 10.1104/pp.109.136762 PMC2675724

[B39] Fukuda H. (1996). Xylogenesis: initiation, progression, and cell death. *Annu. Rev. Plant Physiol. Plant Mol. Biol.* 47 299–325 15012291 10.1146/annurev.arplant.47.1.299

[B40] Fukuda H. (1997). Programmed cell death during vascular system formation. *Cell Death Differ.* 4 684–688 16465280 10.1038/sj.cdd.4400310

[B41] Fukuda H. (2004). Signals that control plant vascular cell differentiation. *Nat. Rev. Mol. Cell Biol.* 5 379–391 15122351 10.1038/nrm1364

[B43] Gartemann K.-H. Abt B. Bekel T. Burger A. Engemann J. Flugel M. (2008). The genome sequence of the tomato-pathogenic *Actinomycete Clavibacter michiganensis* subsp. *michiganensis* NCPPB382 reveals a large island involved in pathogenicity. *J. Bacteriol.* 190 2138–2149 10.1128/JB.01595-07 PMC2258877 18192381

[B42] Gayoso C. Pomar F. Novo-Uzal E. Merino F Martinez de Ilarduya O. (2010). The Ve-mediated resistance response of the tomato to *Verticillium* dahliae involves H2O2, peroxidase and lignins and drives PAL gene expression. *BMC Plant Biol.* 10:232 10.1186/1471-2229-10-232 PMC3095318 20977727

[B44] Genin S. (2010). Molecular traits controlling host range and adaptation to plants in *Ralstonia solanacearum*. *New Phytol.* 187 920–928 20673287 10.1111/j.1469-8137.2010.03397.x

[B45] Gómez-Gómez L. Bauer Z. Boller T. (2001). Both the extracellular leucine-rich repeat domain and the kinase activity of FLS2 are required for flagellin binding and signaling in *Arabidopsis*. *Plant Cell* 13 1155–1163 11340188 PMC135565

[B46] Grimault V. Gélie B. Lemattre M. Prior P. Schmit J. (1994). Comparative histology of resistant and susceptible tomato cultivars infected by *Pseudomonas solanacearum*. *Physiol. Mol. Plant Pathol.* 44 105–123

[B47] Harwood T. D. Tomlinson I. Potter C. A. Knight J. D. (2011). Dutch elm disease revisited: past, present and future management in Great Britain. *Plant Pathol.* 60 545–555

[B48] Hayslett M. Juzwik J. Moltzan B. (2008). Three *Colopterus* beetle species carry the oak wilt fungus to fresh wounds on red oak in Missouri. *Plant Dis.* 92 270–275 10.1094/PDIS-92-2-0270 30769383

[B49] Hayward A. C. (1991). Biology and epidemiology of bacterial wilt caused by *Pseudomonas solanacearum*. *Annu. Rev. Phytopathol.* 29 65–87 18479193 10.1146/annurev.py.29.090191.000433

[B50] Hilaire E. Young S. A. Willard L. H. McGee J. D. Sweat T. Chittoor J. M. (2001). Vascular defense responses in rice: peroxidase accumulation in xylem parenchyma cells and xylem wall thickening. *Mol. Plant Microbe Interact.* 14 1411–1419 11768536 10.1094/MPMI.2001.14.12.1411

[B51] Houterman P. M. Ma L. Van Ooijen G. De Vroomen M. J. Cornelissen B. J. C. Takken F. L. W. (2009). The effector protein Avr2 of the xylem-colonizing fungus *Fusarium oxysporum* activates the tomato resistance protein I-2 intracellularly. *Plant J.* 58 970–978 19228334 10.1111/j.1365-313X.2009.03838.x

[B52] Houterman P. M. Speijer D. Dekker H. L. De Koster C. G. Cornelissen B. J. C. Rep M. (2007). The mixed xylem sap proteome of *Fusarium oxysporum*-infected tomato plants: short communication. *Mol. Plant Pathol.* 8 215–221 20507493 10.1111/j.1364-3703.2007.00384.x

[B53] Huang C.-C. Lindhout P. (1997). Screening for resistance in wild *Lycopersicon* species to *Fusarium oxysporum* f. sp. *lycopersici* race 1 and race 2. *Euphytica* 93 145–153

[B54] Janse J. D. Obradovic A. (2010). *Xylella fastidiosa*: its biology, diagnosis, control and risks. *J. Plant Pathol. * 92 S1.35–S31.47

[B55] Jansen S. Baas P. Gasson P. Lens F. Smets E. (2004). Variation in xylem structure from tropics to tundra: evidence from vestured pits. *Proc. Natl. Acad. Sci. U.S.A.* 101 8833–8837 15163796 10.1073/pnas.0402621101 PMC423281

[B56] Jha G. Rajeshwari R. Sonti R. V. (2005). Bacterial type two secretion system secreted proteins: double-edged swords for plant pathogens. *Mol. Plant Microbe Interact.* 18 891–898 16167759 10.1094/MPMI-18-0891

[B57] Ji X. Lu G. Gai Y. Zheng C. Mu Z. (2008). Biological control against bacterial wilt and colonization of mulberry by an endophytic *Bacillus subtilis* strain. *FEMS Microbiol. Ecol.* 65 565–573 18631174 10.1111/j.1574-6941.2008.00543.x

[B58] Jiang Y. Chen X. Ding X. Wang Y. Chen Q. Song W.-Y. (2012). The XA21 binding protein XB25 is required for maintaining XA21-mediated disease resistance. *Plant J.* 73 814–823 23206229 10.1111/tpj.12076

[B59] Jones J. D. G. Dangl J. L. (2006). The plant immune system. *Nature* 444 323–329 17108957 10.1038/nature05286

[B60] Juzwik J. Harrington T. C. MacDonald W. L. Appel D. N. (2008). The origin of *Ceratocystis fagacearum*, the oak wilt fungus. *Annu. Rev. Phytopathol.* 46 13–26 18680421 10.1146/annurev.phyto.45.062806.094406

[B61] Kawchuk L. M. Hachey J. Lynch D. R. Kulcsar F. Van Rooijen G. Waterer D. R. (2001). Tomato Ve disease resistance genes encode cell surface-like receptors. *Proc. Natl. Acad. Sci. U.S.A.* 98 6511–6515 11331751 10.1073/pnas.091114198 PMC33499

[B62] Klosterman S. J. Atallah Z. K. Vallad G. E. Subbarao K. V. (2009). Diversity, pathogenicity, and management of *Verticillium* species. *Annu. Rev. Phytopathol.* 47 39–61 19385730 10.1146/annurev-phyto-080508-081748

[B63] Klosterman S. J. Subbarao K. V. Kang S. Veronese P. Gold S. E. Thomma B. P. H. J. (2011). Comparative genomics yields insights into niche adaptation of plant vascular wilt pathogens. *PLoS Pathol. * 7:e1002137 10.1371/journal.ppat.1002137 PMC3145793 21829347

[B64] Krishnan H. B. Natarajan S. S. Bennett J. O. Sicher R. C. (2011). Protein and metabolite composition of xylem sap from field-grown soybeans (*Glycine max*). *Planta* 233 921–931 21246215 10.1007/s00425-011-1352-9

[B65] Kunze G. Zipfel C. Robatzek S. Niehaus K. Boller T. Felix G. (2004). The N terminus of bacterial elongation factor Tu elicits innate immunity in *Arabidopsis* plants. *Plant Cell* 16 3496–3507 15548740 10.1105/tpc.104.026765 PMC535888

[B66] Lee S.-W. Han S.-W. Sririyanum M. Park C.-J. Seo Y.-S. Ronald P. C. (2009). A type I-secreted, sulfated peptide triggers XA21-mediated innate immunity. *Science* 326 850–853 19892983 10.1126/science.1173438

[B67] Lee B. M. Park Y. J. Park D. S. Kang H. W. Kim J. G. Song E. S. (2005). The genome sequence of *Xanthomonas oryzae* pathovar *oryzae* KACC10331, the bacterial blight pathogen of rice. *Nucleic Acids Res.* 33 577–586 15673718 10.1093/nar/gki206 PMC548351

[B68] Leubner-Metzger G. Meins F. (1999). “Functions and regulation of plant beta-1,3-glucanases (PR-2),” in *Pathogenesis-Related Proteins in Plants*, eds Datta S. K. Muthukrishnan S. (Boca Raton: CRC Press) 49–76

[B69] Lévesque C. A. Brouwer H. Cano L. Hamilton J. Holt C. Huitema E. (2010). Genome sequence of the necrotrophic plant pathogen *Pythium ultimum* reveals original pathogenicity mechanisms and effector repertoire. *Genome Biol. * 11 R73 10.1186/gb-2010-11-7-r73 PMC2926784 20626842

[B70] Lievens B. Rep M. Thomma B. P. (2008). Recent developments in the molecular discrimination of formae speciales of *Fusarium oxysporum*. *Pest Manag. Sci.* 64 781–788 18335459 10.1002/ps.1564

[B71] López-Escudero F. Mercado-Blanco J. (2011). *Verticillium* wilt of olive: a case study to implement an integrated strategy to control a soil-borne pathogen. *Plant Soil* 344 1–50

[B72] Ma L.-J. van der Does H. C. Borkovich K. A. Coleman J. J. Daboussi M.-J. Di Pietro A. (2010). Comparative genomics reveals mobile pathogenicity chromosomes in Fusarium. *Nature* 464 367–373 20237561 10.1038/nature08850 PMC3048781

[B73] Markakis E. A. Tjamos S. E. Chatzipavlidis I. Antoniou P. P. Paplomatas E. J. (2008). Evaluation of compost amendments for control of vascular wilt diseases. *J. Phytopathol.* 156 622–627

[B74] Martin F. N. Loper J. E. (1999). Soilborne plant diseases caused by *Pythium* spp.: ecology, epidemiology, and prospects for biological control. *Crit. Rev. Plant Sci.* 18 111–181

[B75] Martïn J. A. Solla A. Domingues M. R. Coimbra M. A. Gil L. (2008). Exogenous phenol increase resistance of *Ulmus minor* to Dutch elm disease through formation of suberin-like compounds on xylem tissues. *Environ. Exp. Bot.* 64 97–104

[B76] Marzluf G. (1997). Genetic regulation of nitrogen metabolism in the fungi. *Microbiol. Mol. Biol. Rev.* 61 17–32 9106362 10.1128/mmbr.61.1.17-32.1997 PMC232598

[B77] McCully M. E. (2001). Niches for bacterial endophytes in crop plants: a plant biologist’s view. *Funct. Plant Biol.* 28 983–990

[B78] Michielse C. B. Rep M. (2009). Pathogen profile update: *Fusarium oxysporum*. *Mol. Plant Pathol.* 10 311–324 19400835 10.1111/j.1364-3703.2009.00538.x PMC6640313

[B79] Möbius N. Hertweck C. (2009). Fungal phytotoxins as mediators of virulence. *Curr. Opin. Plant Biol.* 12 390–398 19608453 10.1016/j.pbi.2009.06.004

[B80] Mollenhauer H. H. Hopkins D. L. (1974). Ultrastructural study of Pierce’s disease bacterium in grape xylem tissue. *J. Bacteriol.* 119 612–618 4855307 10.1128/jb.119.2.612-618.1974 PMC245648

[B81] Moser J. C. Konrad H. Blomquist S. R. Kirisits T. (2010). Do mites phoretic on elm bark beetles contribute to the transmission of Dutch elm disease? *Naturwissenschaften* 97 219–227 19967528 10.1007/s00114-009-0630-x

[B82] Nadarasah G. Stavrinides J. (2011). Insects as alternative hosts for phytopathogenic bacteria. *FEMS Microbiol. Rev.* 35 555–575 21251027 10.1111/j.1574-6976.2011.00264.x

[B83] Nakamura S.-I. Akiyama C. Sasaki T. Hattori H. Chino M. (2008). Effect of cadmium on the chemical composition of xylem exudate from oilseed rape plants (*Brassica napus* L.). *Soil Sci. Plant Nutr.* 54 118–127

[B84] Newcombe G. Robb J. (1988). The function and relative importance of the vascular coating response in highly resistant, moderately resistant and susceptible alfalfa infected by *Verticillium albo-atrum*. *Physiol. Mol. Plant Pathol.* 33 47–58

[B85] Nieminen K. M. Kauppinen L. Helariutta Y. (2004). A weed for wood? *Arabidopsis* as a genetic model for xylem development. *Plant Physiol.* 135 653–659 15208411 10.1104/pp.104.040212 PMC514101

[B86] Niño-Liu D. O. Ronald P. C. Bogdanove A. J. (2006). *Xanthomonas oryzae* pathovars: model pathogens of a model crop. *Mol. Plant Pathol.* 7 303–324 20507449 10.1111/j.1364-3703.2006.00344.x

[B87] Oliver J. P. Castro A. Gaggero C. Cascón T. Schmelz E. A. Castresana C. (2009). Pythium infection activates conserved plant defense responses in mosses. *Planta* 230 569–579 19551405 10.1007/s00425-009-0969-4

[B88] Palmer C. S. Saleeba J. A. Lyon B. R. (2005). Phytotoxicity on cotton ex-plants of an 18.5 kDa protein from culture filtrates of *Verticillium dahliae*. *Physiol. Mol. Plant Pathol.* 67 308–318

[B89] Park C.-J. Bart R. Chern M. Canlas P. E. Bai W. Ronald P. C. (2010a). Overexpression of the endoplasmic reticulum chaperone BiP3 regulates XA21-mediated innate immunity in rice. *PLoS ONE* 5:e9262 10.1371/journal.pone.0009262 PMC2822859 20174657

[B90] Park C. J. Han S. W. Chen X. Ronald P. C. (2010b). Elucidation of XA21-mediated innate immunity. *Cell. Microbiol.* 12 1017–1025 20590657 10.1111/j.1462-5822.2010.01489.x PMC2906629

[B91] Passardi F. Cosio C. Penel C. Dunand C. (2005). Peroxidases have more functions than a Swiss army knife. *Plant Cell Rep.* 24 255–265 15856234 10.1007/s00299-005-0972-6

[B92] Pérez-Donoso A. G. Greve L. C. Walton J. H. Shackel K. A. Labavitch J. M. (2007). *Xylella fastidiosa* infection and ethylene exposure result in xylem and water movement disruption in grapevine shoots. *Plant Physiol.* 143 1024–1036 17189331 10.1104/pp.106.087023 PMC1803717

[B93] Pérez-Donoso A. G. Sun Q. Roper M. C. Greve L. C. Kirkpatrick B. Labavitch J. M. (2010). Cell wall-degrading enzymes enlarge the pore size of intervessel pit membranes in healthy and *Xylella fastidiosa*-infected grapevines. *Plant Physiol.* 152 1748–1759 20107028 10.1104/pp.109.148791 PMC2832268

[B94] Powney R. Smits T. H. M. Sawbridge T. Frey B. Blom J. Frey J. E. (2011). Genome sequence of an *Erwinia amylovora* strain with pathogenicity restricted to Rubus plants. *J. Bacteriol.* 193 785–786 21131493 10.1128/JB.01352-10 PMC3021219

[B95] Purcell A. H. Hopkins D. L. (1996). Fastidious xylem-limited bacterial plant pathogens. *Annu. Rev. Phytopathol.* 34 131–151 15012538 10.1146/annurev.phyto.34.1.131

[B96] Qian W. Jia Y. Ren S.-X. He Y.-Q. Feng J.-X. Lu L.-F. (2005). Comparative and functional genomic analyses of the pathogenicity of phytopathogen *Xanthomonas campestris* pv. *campestris*. *Genome Res.* 15 757–767 15899963 10.1101/gr.3378705 PMC1142466

[B97] Qin G. M. Vallad G. E. Subbarao K. V. (2008). Characterization of *Verticillium dahliae* and *V. tricorpus* isolates from lettuce and artichoke. *Plant Disease* 92 69–77 10.1094/PDIS-92-1-0069 30786364

[B98] Rahman M. A. Abdullah H. Vanhaecke M. (1999). Histopathology of susceptible and resistant *Capsicum annuum* cultivars infected with *Ralstonia solanacearum*. *J. Phytopathol.* 147 129–140

[B99] Rep M. Dekker H. L. Vossen J. H. De Boer A. D. Houterman P. M. De Koster C. G. (2003). A tomato xylem sap protein represents a new family of small cysteine-rich proteins with structural similarity to lipid transfer proteins. *FEBS Lett.* 534 82–86 12527365 10.1016/s0014-5793(02)03788-2

[B100] Rep M. Dekker H. L. Vossen J. H. De Boer A. D. Houterman P. M. Speijer D. (2002). Mass spectrometric identification of isoforms of PR proteins in xylem sap of fungus-infected tomato. *Plant Physiol.* 130 904–917 12376655 10.1104/pp.007427 PMC166617

[B101] Reusche M. Thole K. Janz D. Truskina J. Rindfleisch S. Drübert C. (2012). *Verticillium* infection triggers VASCULAR-RELATED NAC DOMAIN7-dependent de novo xylem formation and enhances drought tolerance in *Arabidopsis*. *Plant Cell* 24 3823–3837 23023171 10.1105/tpc.112.103374 PMC3480305

[B102] Rey P. Benhamou N. Tirilly Y. (1998). Ultrastructural and cytochemical investigation of asymptomatic infection by *Pythium* spp. *Phytopathology* 88 234–244 18944970 10.1094/PHYTO.1998.88.3.234

[B103] Roncero M. I. G. Hera C. Ruiz-Rubio M. Garcïa Maceira F. I. Madrid M. P. Caracuel Z. (2003). *Fusarium* as a model for studying virulence in soilborne plant pathogens. *Physiol. Mol. Plant Pathol.* 62 87–98

[B104] Roper M. C. (2011). Pantoea stewartii subsp. stewartii: lessons learned from a xylem-dwelling pathogen of sweet corn. *Mol. Plant Pathol.* 12 628–637 10.1111/j.1364-3703.2010.00698.x PMC6640275 21726365

[B105] Salanoubat M. Genin S. Artiguenave F. Gouzy J. Mangenot S. Arlat M. (2002). Genome sequence of the plant pathogen *Ralstonia solanacearum*. *Nature* 415 497–502 11823852 10.1038/415497a

[B106] Santhanam P. van Esse H. P. Albert I. Faino L. Nürnberger T Thomma B. P. H. J. (2013). Evidence for functional diversification within a fungal NEP1-like protein family. *Mol. Plant Microbe Interact.* 26 278–286 23051172 10.1094/MPMI-09-12-0222-R

[B107] Scheffer R. J. Voeten J. G. W. F. Guries R. P. (2008). Biological control of Dutch elm disease. *Plant Dis.* 92 192–200 10.1094/PDIS-92-2-0192 30769380

[B108] Schumann G. L D’Arcy C. J. (2010). *Essential Plant Pathology*. St. Paul, MN: APS Press

[B109] Sebaihia M. Bocsanczy A. M. Biehl B. S. Quail M. A. Perna N. T. Glasner J. D. (2010). Complete genome sequence of the plant pathogen *Erwinia amylovora* strain ATCC 49946. *J. Bacteriol.* 192 2020–2021 20118253 10.1128/JB.00022-10 PMC2838050

[B110] Siebrecht S. Herdel K. Schurr U. Tischner R. (2003). Nutrient translocation in the xylem of poplar – diurnal variations and spatial distribution along the shoot axis. *Planta* 217 783–793 12721678 10.1007/s00425-003-1041-4

[B111] Simpson A. J. Reinach F. C. Arruda P. Abreu F. A. Acencio M. Alvarenga R. (2000). The genome sequence of the plant pathogen *Xylella fastidiosa*. *Nature* 406 151–159 10910347 10.1038/35018003

[B112] Smits T. H. Rezzonico F. Kamber T. Blom J. Goesmann A. Frey J. E. (2010). Complete genome sequence of the fire blight pathogen *Erwinia amylovora* CFBP 1430 and comparison to other *Erwinia* spp. *Mol. Plant Microbe Interact.* 23 384–393 20192826 10.1094/MPMI-23-4-0384

[B113] Song W. Y. Wang G. L. Chen L. L. Kim H. S. Pi L. Y. Holsten T. (1995). A receptor kinase-like protein encoded by the rice disease resistance gene, Xa21. *Science* 270 1804–1806 8525370 10.1126/science.270.5243.1804

[B114] Spadaro D. Gullino M. L. (2005). Improving the efficacy of biocontrol agents against soilborne pathogens. *Crop Prot.* 24 601–613

[B115] Stipanovic R. D. Puckhaber L. S. Liu J. Bell A. A. (2011). Phytotoxicity of fusaric acid and analogs to cotton. *Toxicon* 57 176–178 20955724 10.1016/j.toxicon.2010.10.006

[B116] Street P. F. S. Robb J. Ellis B. E. (1986). Secretion of vascular coating components by xylem parenchyma cells of tomatoes infected with *Verticillium albo-atrum*. *Protoplasma* 132 1–11

[B117] Suárez-Estrella F. Vargas-Garcïa C. López M. J. Capel C. Moreno J. (2007). Antagonistic activity of bacteria and fungi from horticultural compost against *Fusarium oxysporum* f. sp. *melonis*.* Crop Prot.* 26 46–53

[B118] Sun Q. H. Hu J. Huang G. X. Ge C. Fang R. X. He C. Z. (2005). Type-II secretion pathway structural gene xpsE, xylanase- and cellulase secretion and virulence in *Xanthomonas oryzae* pv. *oryzae. Plant Pathol.* 54 15–21

[B119] Takken F. Rep M. (2010). The arms race between tomato and *Fusarium oxysporum*. *Mol. Plant Pathol.* 11 309–314 20447279 10.1111/j.1364-3703.2009.00605.x PMC6640361

[B120] Talboys P. W. (1972). Resistance to vascular wilt fungi. *Proc. R. Soc. Lond. B Biol. Sci.* 181 319–332

[B121] Temple B. Horgen P. A. (2000). Biological roles for cerato-ulmin, a hydrophobin secreted by the elm pathogens, *Ophiostoma ulmi* and *O. novo-ulmi*. * Mycologia* 92 1–9

[B122] Tjamos E. C. Beckman C. H. (1989). *Vascular Wilt Diseases of Plants: Basic Studies and Control (NATO ASI Series H: Cell Biology)*. Berlin: Springer-Verlag

[B123] Tsuda K. Kosaka Y. Tsuge S. Kubo Y. Horino O. (2001). Evaluation of the endophyte *Enterobacter cloacae* SM10 isolated from spinach roots for biological control against *Fusarium* wilt of spinach. *J. Gen. Plant Pathol.* 67 78–84

[B124] Tyree M. T. Zimmermann M. H. (2002). *Xylem Structure and the Ascent of Sap*. Berlin: Springer

[B125] van Esse H. P. Fradin E. F. de Groot P. J. de Wit P. J. G. M. Thomma B. P. H. J. (2009). Tomato transcriptional responses to a foliar and a vascular fungal pathogen are distinct. *Mol. Plant Microbe Interact.* 22 245–258 19245319 10.1094/MPMI-22-3-0245

[B126] van Loon L. C. Rep M Pieterse C. M. J. (2006). Significance of inducible defense-related proteins in infected plants. *Annu. Rev. Phytopathol.* 44 135–162 16602946 10.1146/annurev.phyto.44.070505.143425

[B127] Vicente J. G. Conway J. Roberts S. J. Taylor J. D. (2001). Identification and origin of *Xanthomonas campestris* pv. *campestris* races and related pathovars. *Phytopathology* 91 492–499 10.1094/PHYTO.2001.91.5.492 18943594

[B128] Wang G. Ellendorff U. Kemp B. Mansfield J. W. Forsyth A. Mitchell K. (2008). A genome-wide functional investigation into the roles of receptor-like proteins in *Arabidopsis*. *Plant Physiol.* 147 503–517 18434605 10.1104/pp.108.119487 PMC2409048

[B129] Wang J. Y. Cai Y. Gou J. Y. Mao Y. B. Xu Y. H. Jiang W. H. (2004). VdNEP, an elicitor from *Verticillium dahliae*, induces cotton plant wilting. *Appl. Environ. Microbiol.* 70 4989–4995 15294839 10.1128/AEM.70.8.4989-4995.2004 PMC492334

[B130] Williams J. S. Hall S. A. Hawkesford M. J. Beale M. H. Cooper R. M. (2002). Elemental sulfur and Thiol accumulation in tomato and defense against a fungal vascular pathogen. *Plant Physiol.* 128 150–159 11788760 PMC148958

[B131] Xu R.-Q. Blanvillain S. Feng J.-X. Jiang B.-L. Li X.-Z. Wei H.-Y. (2008). AvrACXcc8004, a type III effector with a leucine-rich repeat domain from *Xanthomonas campestris* pathovar *campestris* confers avirulence in vascular tissues of *Arabidopsis thaliana* ecotype Col-0. *J. Bacteriol.* 190 343–355 17951377 10.1128/JB.00978-07 PMC2223733

[B132] Yadeta K. A. Hanemian M. Smit P. Hiemstra J. A. Pereira A. Marco Y. (2011). The *Arabidopsis thaliana* DNA-binding protein AHL19 mediates *Verticillium* wilt resistance. *Mol. Plant Microbe Interact.* 24 1582–1591 21864046 10.1094/MPMI-04-11-0090

[B133] Ye Z. H. (2002). Vascular tissue differentiation and pattern formation in plants. *Annu. Rev. Plant Biol.* 53 183–202 12221972 10.1146/annurev.arplant.53.100301.135245

[B134] Yokoyama R. Nishitani K. (2006). Identification and characterization of *Arabidopsis thaliana* genes involved in xylem secondary cell walls. *J. Plant Res.* 119 189–194 16552477 10.1007/s10265-006-0261-7

[B135] Zhang J. Elo A. Helariutta Y. (2011). *Arabidopsis* as a model for wood formation. *Curr. Opin. Biotechnol.* 22 293–299 21144727 10.1016/j.copbio.2010.11.008

[B136] Zhang Z. Fradin E. Jonge R. van Esse H. P. Smit P. Liu C.-M. (2013). Optimized agroinfiltration and virus-induced gene silencing to study Ve1-mediated *Verticillium* resistance in tobacco. *Mol. Plant Microbe Interact.* 26 182–190 22991998 10.1094/MPMI-06-12-0161-R

[B137] Zhou B.-J. Jia P.-S. Gao F. Guo H.-S. (2012). Molecular characterization and functional analysis of a necrosis- and ethylene-inducing, protein-encoding gene family from *Verticillium dahliae*. *Mol. Plant Microbe Interact.* 25 964–975 22414440 10.1094/MPMI-12-11-0319

[B138] Zipfel C. Kunze G. Chinchilla D. Caniard A. Jones J. D. G. Boller T. (2006). Perception of the bacterial PAMP EF-Tu by the receptor EFR restricts *Agrobacterium*-mediated transformation. *Cell* 125 749–760 16713565 10.1016/j.cell.2006.03.037

